# The In Vitro Simulated Gastrointestinal Digestion Affects the Bioaccessibility and Bioactivity of *Beta vulgaris* Constituents

**DOI:** 10.3390/foods12020338

**Published:** 2023-01-11

**Authors:** Marta Igual, Ângela Fernandes, Maria Inês Dias, José Pinela, Purificación García-Segovia, Javier Martínez-Monzó, Lillian Barros

**Affiliations:** 1 Food Investigation and Innovation Group, Food Technology Department, Universitat Politècnica de València, Camino de Vera s/n, 46022 Valencia, Spain; 2 Centro de Investigação de Montanha (CIMO), Instituto Politécnico de Bragança, Campus de Santa Apolónia, 5300-253 Bragança, Portugal; 3 Laboratório Associado Para a Sustentabilidade e Tecnologia em Regiões de Montanha (SusTEC), Instituto Politécnico de Bragança, Campus de Santa Apolónia, 5300-253 Bragança, Portugal

**Keywords:** beetroot, betacyanins, betanin, macrominerals, trace elements, quinic acid, simulated gastrointestinal digestion, antioxidant activity, antihemolytic activity

## Abstract

Beetroot (*Beta vulgaris* L.) is an important root vegetable crop and a valuable food source of micronutrients and bioactive constituents. In this study, the bioaccessibility of minerals, organic acids, and betacyanins in beetroot powder during simulated gastrointestinal digestion was investigated, as well as the antioxidant activity of the final fractions of each phase of the process. Mineral elements were analyzed by inductively coupled plasma optical emission spectroscopy (ICP-OES), organic acids by ultra-fast liquid chromatography with photodiode array detection (UFLC-PDA), and betacyanins by liquid chromatography with diode-array detection and mass spectrometry (HPLC-DAD-ESI/MS*^n^*). The oxidative hemolysis inhibition assay was used to assess the ex vivo antioxidant activity. The bioaccessibility of minerals at the end of gastrointestinal digestion ranged from 43 to 65%, depending on the mineral element. Among these, Mg was the most bioaccessible, while Ca and Fe had the lowest bioaccessibility. For organic acids, a major release during digestion was observed for quinic acid. It was also found that betanin (the major betalain in beetroot) was highly unstable during the digestion process, probably due to its hydrophilic nature, which agreed with the significant (*p* < 0.05) decrease in antioxidant/antihemolytic activity. These results suggest that beetroot antioxidant compounds are unstable under gastrointestinal conditions, and could be useful for future development of novel and more stable beetroot food formulations.

## 1. Introduction

Beetroot (*Beta vulgaris* L.) is an herbaceous plant in the Betoideae subfamily of the Amaranthaceae family, and it is the most well-known and economically important crop of the Caryophyllales order [1]. The deep red-colored beetroots are the most popular for human consumption, but this species comprises cultivars with bulb colors ranging from yellow to red. This root vegetable is rich in carbohydrates, fiber, micronutrients (including mineral elements and vitamins), and other bioactive compounds such as carotenoids, flavonoids, and betalains, which have been associated with a wide range of biological properties and health-promoting effects [2,3,4,5,6]. Betalains are the main group of phenolic compounds in beetroot, and can be subgrouped into yellow-orange betaxanthins and red-purple betacyanins [7]. Thus, the phytochemical diversity of beetroot makes it a potential source of nutraceuticals that can be used to formulate functional foods and beverages.

Beetroot exploitation for food and nutraceutical application has been investigated by several researchers and the food industry due to its intense color and nutritional properties. The deep red-colored beets are used as food, being consumed both raw and cooked, and are also processed into juices and ready-to-eat, frozen, and dehydrated products. In addition, beetroot pigments can be considered as an alternative to artificial colorants, with the potential to meet the growing demand of the food sector for natural colorants, and also as a marketing strategy [8,9]. At the same time, consumers are favoring green consumerism with less artificial additives added to food products, as natural coloring molecules are generally regarded as safe substances for human consumption [10]. Therefore, natural colorants are more anticipated than their artificial counterparts for industrial food application as additives. Indeed, some artificial colorants can have negative effects on human health, cause allergic reactions, hyperactivity in sensitive children, or have carcinogenic effects upon medium and long-term exposure [11,12]. On the other hand, the water solubility of natural color molecules facilitates their incorporation into aqueous food systems. These natural food colorants are more attractive and have additional positive health effects due to their biological activity. However, the overall nutrient content of foods does not provide complete details about their nutritional quality, and nutrient bioaccessibility should be a key parameter to consider [13]. For this purpose, the digestive process can be simulated using in vitro methodologies, which evaluate the bioaccessibility or concentration of selected nutrients along the digestive tract. These methods are relatively fast and do not harm the animal or human subjects required for in vivo tests [14].

Although there are several studies on the nutritional and functional compounds of beetroot [6,15,16], there is still a need to investigate the digestibility and bioaccessibility of its constituents in order to understand their fate in the human body. Furthermore, studies on bioaccessibility of food constituents have received increasing importance due to the existence of micronutrient deficiencies associated with health issues [17]. Therefore, this work was carried out to evaluate the bioaccessibility of mineral elements, organic acids, and betacyanins from beetroot powder during its simulated gastrointestinal digestion, as well as the in vitro antioxidant activity of extracts resulting from the different stages of the process. For this, the INFOGEST standardized method for sequential gastrointestinal food digestion was implemented with the latest improvement modifications [18,19].

## 2. Materials and Methods

### 2.1. Plant Material and Sample Preparation

Beetroot powder produced by spray-drying with maltodextrin was supplied by Manufacturas Ceylan S.L., Spain. For this work, the powdered samples were rehydrated with bi-distilled water to reach the same water content as the fresh root vegetable (an average value of 85 g/100 g fw was taken according to Kaur and Singh [2] and Kale et al. [15]) and immediately subjected to in vitro simulated gastrointestinal digestion. Maltodextrin is a carrier widely used in the food industry due to its high water solubility, low viscosity, digestibility, and colorless solutions. Thus, encapsulated compounds can be quickly released during digestion, leaving them exposed to gastrointestinal conditions [20].

### 2.2. Simulated Gastrointestinal Digestion

**Figure 1:**
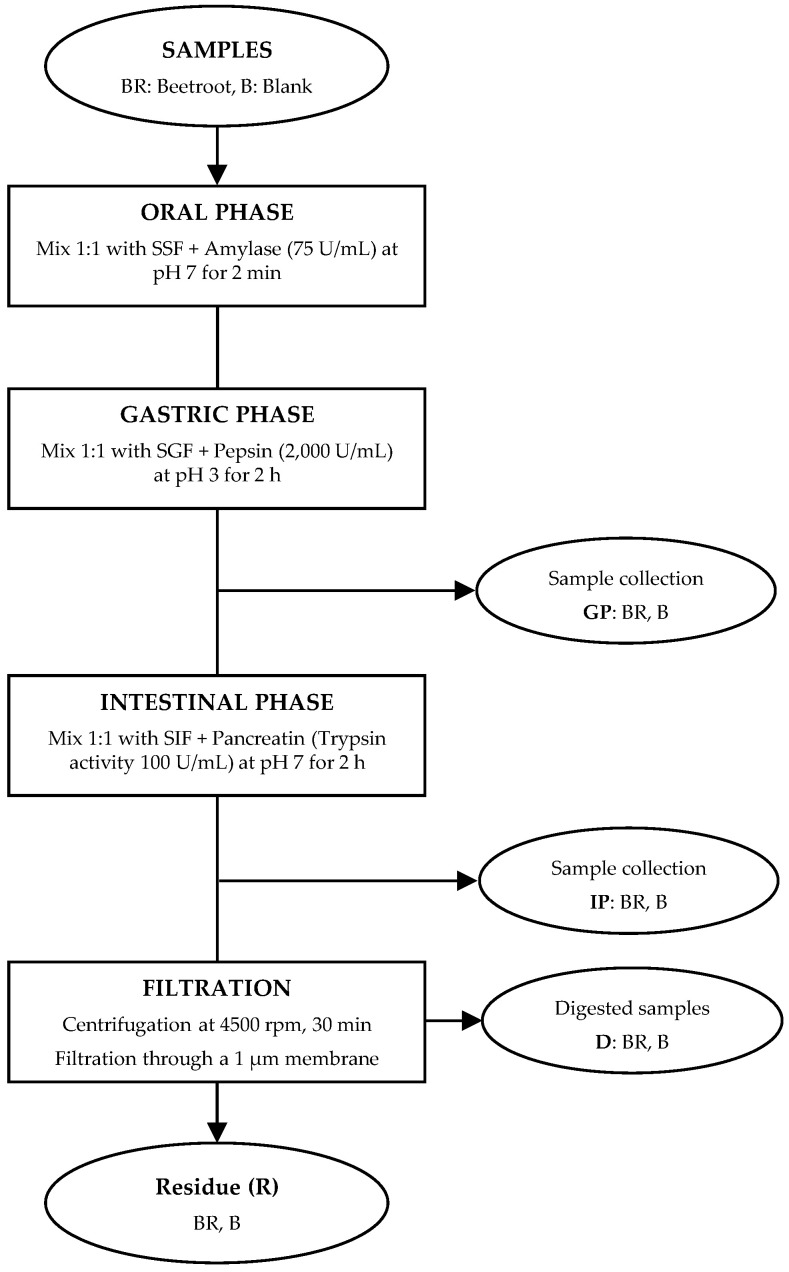
Flow diagram of the static in vitro model used for beetroot digestion. After mixing the beetroot sample with simulated salivary fluid (SSF) and amylase at pH 7 for 2 min, the oral bolus was mixed with simulated gastric fluid (SGF), pepsin, and gastric lipase at pH 3 for 2 h to obtain the gastric phase (GP). Then, the intestinal phase (IP) was obtained by mixing the gastric chime with simulated intestinal fluid (SIF) and pancreatin at pH 7 for 2 h. Finally, the mixture was centrifugated and filtered to obtain the digested (D) sample. More details are provided in Table S1.

Sample digestibility was evaluated using the standardized static in vitro digestion method for foods proposed by the INFOGEST^®^ network [18] and Brodkorb et al. [19], which is an international consensus method to simulate gastrointestinal digestion. As shown in Figure 1, this protocol includes the following four steps: oral phase (GP, pH 7), gastric phase (GP, pH 3), intestinal phase (IP, pH 7), and digested (D) sample. More details on the steps of this method are described in Table S1. Both beetroot (BR) and a blank (B) were digested in vitro, and an aliquot was collected from each phase and freeze-dried with a protease inhibitor (Pefabloc SC, Sigma-Aldrich, St. Louis, MO, USA). The concentration of the enzymes used in the assay was estimated according the activity certificated by the manufacturer. The simulated fluids were prepared according to Minekus et al. [13]. All samples (BR, B, GP, IP, and D) were analyzed in triplicate for mineral, organic acid, and betacyanin contents, as well as antioxidant activity. The water and reagents used in the simulated digestion were analyzed to correct the result obtained for each fraction. In addition, in vitro digestibility (IVD) (%) was calculated as previously described [21].

### 2.3. Measurement of Water Content and Activity

The water content (%, *w/w*) of the powder and rehydrated beetroot samples was determined in triplicate by vacuum oven drying at 60 °C until a constant weight was achieved, following the procedures of AOAC International [22]. The water activity (*a*_w_) of the beetroot powder was assessed using an AquaLab PRE LabFerrer meter (Pullman, DC, USA).

### 2.4. Mineral Content Determination

Undigested and digested beetroot samples were incinerated in a microwave oven at 550 °C for 24 h, and the ash content was determined gravimetrically. The incineration residue was then extracted with HCl (50%, *v/v*) and HNO_3_ (50%, *v/v*) [23]. The analysis was performed using an inductively coupled plasma optical emission spectrometer (700 Series ICP-OES) [24], equipped as described in Table S2. The levels of macrominerals and trace elements quantified were expressed as mg per 100 g fresh weight (fw) of beetroot.

### 2.5. Organic Acid Analysis

Organic acids were extracted from undigested and digested beetroot samples as previously described by Pereira et al. [25] and then analyzed in an ultra-fast liquid chromatography (UFLC) system coupled to a photodiode array detector (215 nm was the wavelength selected for the analysis). The detected compounds were identified by comparing the retention time and the UV-vis spectrum of the sample peaks with those of commercial standards of oxalic, quinic, malic, shikimic, citric acid, succinic, and fumaric acids (purchased from Sigma-Aldrich, St. Louis, MO, USA) in Figures S1 and S2, and then quantified (mg per 100 g fw of beetroot) by comparing peak areas with calibration curves constructed with the same standards. More details about the equipment and chromatographic method are described in Table S2.

### 2.6. Betacyanin Analysis

Undigested and digested samples were dissolved in water to a final concentration of 50 mg/mL and analyzed by HPLC-DAD-ESI/MS*^n^*, as previously described [26]. Double detection was carried out in the diode array detector (535 nm was the wavelength selected for the analysis) and in a Linear Ion Trap LTQ XL MS mass spectrometer. More details on the equipment and chromatographic conditions are described in Table S2. The identification of the detected betacyanins was conducted by comparing the chromatographic information with the available data from the literature. For compound quantification (mg per 100 g fw of beetroot), the following calibration curve was constructed using gomphrenin III that had previously been isolated by the authors from *Gomphrena globosa* L.: *y* = 14670*x* − 19725, *r*^2^ = 0.9997, limit of detection = 0.78 µg/mL, and limit of quantification = 1.97 µg/mL [26].

### 2.7. Antioxidant Activity Evaluation

The in vitro antioxidant activity of undigested and digested samples was assessed by the oxidative hemolysis inhibition method previously described by the authors [27]. Briefly, a sheep red blood cell (RBC) solution (2.8%, *v/v*) was mixed with either beetroot sample dissolved in PBS, PBS solution (negative control), trolox (positive control), or distilled water (baseline). After 10 min incubation at 37 °C with shaking, the free radical generator 2,2’-azobis(2-amidinopropane) dihydrochloride (160 mM) was added, and the decrease in the optical density was monitored kinetically at 690 nm in a microplate reader (Bio-Tek Instruments, ELX800). The results were analyzed using GraphPad Prism^®^ 8 and expressed in mg of Trolox equivalent per 100 g fw of beetroot, considering a 60 min Δ*t*.

### 2.8. Statistical Analysis

Differences between undigested and digested samples were evaluated through an analysis of variance (ANOVA) at a 95% confidence level. A Pearson’s correlation analysis between antioxidant capacity and the studied components was also performed. These statistical analyses were performed using Statgraphics (Centurion XVII Software, version 17.2.04).

## 3. Results and Discussion

### 3.1. Water Content and Activity

The mean values and respective standard deviation obtained for the water content and water activity (*a*_w_) of the studied beetroot powder were 4.06 ± 0.05% and 0.285 ± 0.003, respectively. To simulate gastrointestinal digestion, the beetroot powder was initially rehydrated to 84.97 ± 0.02%. The in vitro digestibility (IVD) analysis reproduced the chemical–enzymatic catalysis to which the sample was submitted in the proximal tract of the monogastric digestive system [28]. The IVD result of the studied beetroot sample was 99.65 ± 0.04%. Other authors who used a different static in vitro digestion method for spirulina observed values slightly lower than those obtained in the present work [21].

### 3.2. Mineral Elements

**Figure 2:**
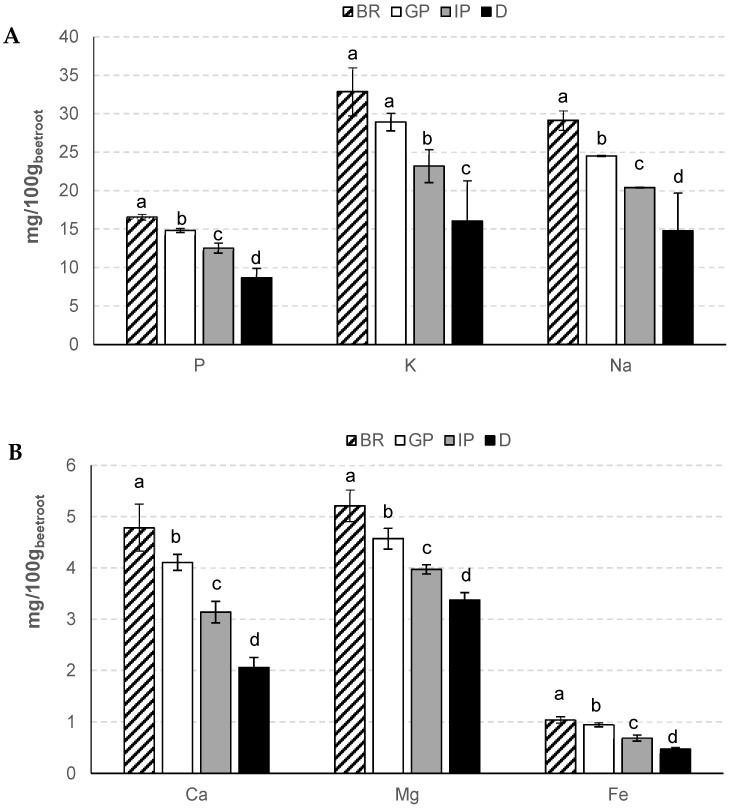
Contents (mg/100 g fw of beetroot) of P, K, and Na (**A**) and of Ca, Mg, and Fe (**B**) in beetroot (BR), gastric phase (GP), intestinal phase (IP) and digested (D) sample. For each element, different letters indicate significant differences (*p* < 0.05) between samples.

Figure 2 shows the mineral content in beetroot before and after each gastrointestinal digestion phase. The mineral values for the studied beetroot powder sample, obtained from spray-dried beetroot juice, were lower than those (256 mg/100 g P, 3054 mg/100 g K, 413 mg/100 g Ca, 218 mg/100 g Mg, and 912 mg/100 g Fe) reported for beetroot juice [16]. In addition, the trace elements Cu, Zn, Se, and Mn were not detected in the studied sample. After in vitro gastrointestinal digestion, the mineral content decreased significantly (*p* < 0.05), since a portion was not accessible for absorption by the digestive tract.

The bioavailability of mineral elements in food can be affected by the presence of antinutritional factors such as oxalates, phytates, tannins, and saponins, which can cause complexation, inhibition, and binding of these dietary elements, thus increasing mineral balance and decreasing their bioaccessibility [29]. As can be seen in Figure 2, when relating the mineral content in each digestion phase with the total amount present in beetroot (BR), it appears that the percentage of mineral bioaccessibility ranged from 85 to 90% in the gastric phase (GP), from 65 to 76% in the intestinal phase (IP), and from 43 to 65% in the digested (D) sample, depending on the quantified mineral. In fact, for each element, there were significant differences (*p* < 0.05) in their contents across the studied digestion stages. For the digested sample, P, K, and Na showed similar bioaccessible content with respect to BR (Figure 2A). The bioaccessibility of Mg was superior to that of the other studied minerals (Figure 2B). Uribe-Wandurraga et al. [24] and Vitali et al. [30] also observed this trend in microalgae-enriched cookies and whole grain tea biscuits, respectively, with Mg bioaccessibility values of ≈70 and 75%, respectively. In contrast, the bioaccessibility values of Ca and Fe were the lowest among the minerals studied in the digested sample. Soluble oxalates can chelate minerals, forming insoluble salts such as calcium oxalate [29]. In the digested sample, the bioaccessibility values of Ca and Fe, as percentages, were 43% and 46%, respectively (Figure 2B). This decrease in bioaccessibility may be related to antinutritional factors such as oxalate (or oxalic acid), which was detected in the studied sample and has been described in high concentrations in beetroot juice (60–70 mg/100 mL) [31].

### 3.3. Organic Acids

**Figure 3:**
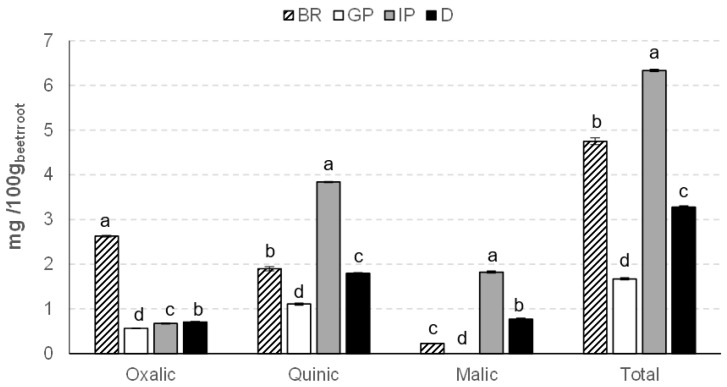
Organic acids content (mg/100 g fw of beetroot) in beetroot (BR), gastric phase (GP), intestinal phase (IP), and digested (D) sample. For each organic acid, different letters indicate significant differences (*p* < 0.05) between samples.

The organoleptic properties of fruits and vegetables, such as taste, color, and aroma, are strongly influenced by organic acids [32,33]. In addition, these constituents indirectly affect the phenolic metabolism by altering pH, and act as precursors to phenolic and flavor compounds [34]. Humans can also benefit from the ingestion of these food constituents. The organic acid content of the beetroot powder before and after each gastrointestinal phase can be observed in Figure 3. Oxalic acid was the most abundant in the analyzed beetroot sample, in agreement with the observations of other authors [35], followed by quinic and malic acids. The chromatograms of the four samples are shown in Figure S3. At the end of digestion, the total organic acids showed 69% bioaccessibility. However, oxalic acid bioaccessibility at the end of gastrointestinal digestion was 27%. The final total organic acid content accessible for absorption by the human body was about 3.27 mg/100 g fw of beetroot. The highest contribution of this value was quinic acid (≈1.79 mg/100 g fw of beetroot). During the intestinal phase (IP), a release of quinic acid occurred, probably from the digestion of phenolic acids such as chlorogenic acid, since other authors have showed that quinic and caffeic acids are released from chlorogenic acid by in vitro human digestion [36]. The malic acid content fluctuated according to the different stages of digestion. At the end of the digestive process, the malic acid content was significantly higher than at the beginning (*p* < 0.05), a behavior that has also been observed by other authors in cranberry juices [37].

### 3.4. Betacyanins

**Figure 4:**
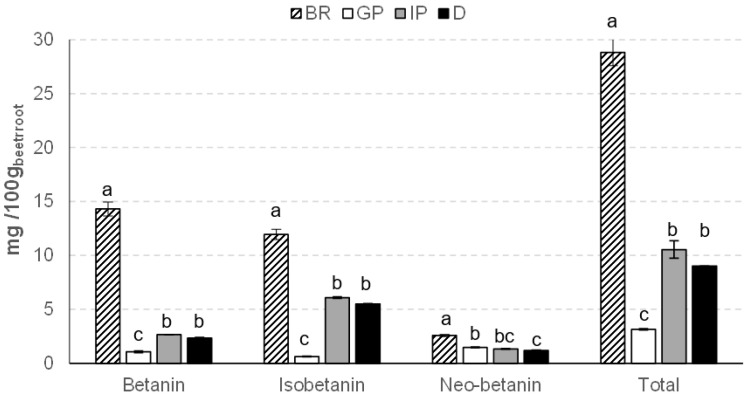
Betacyanins content (mg/100 g fw of beetroot) in beetroot (BR), gastric phase (GP), intestinal phase (IP), and digested (D) sample. For each betacyanin compound, different letters indicate significant differences (*p* < 0.05) between samples.

Betalains are water-soluble pigments that can be grouped into red-colored betacyanins and yellow-colored betaxanthins [38]. The stability of these bioactive compounds is affected by several factors, including their chemical structure, pH, water activity, and enzymes of the medium [7]. The chromatographic data used to identify the betacyanins detected in the beetroot samples are presented in Table 1, and the HPLC chromatographic profile and the UV-vis and mass spectra are shown in Figures S4 and S5, respectively. These betalain compounds (betanin, isobetanin, and neobetanin) were tentatively identified by comparing the chromatographic information with data from the literature [39,40]. Figure 4 shows the mean values and standard deviations of betacyanin content in beetroot before and after gastrointestinal digestion. The total betacyanin content in beetroot was approximately 28.8 mg/100 g fw of beetroot. Betanin was the dominant betalain in the sample, as reported by other authors [41]. This functional constituent was highly unstable at the end of gastrointestinal digestion and showed a bioaccessibility of 16.2%, which may be related to its hydrophilic nature. Meanwhile, a final betanin loss of 83.8% was observed at the end of gastrointestinal digestion due to the possible binding of betacyanins to insoluble material, including protein aggregates formed during digestion [42]. The betanin bioaccessibility value was similar to the result obtained by Montiel-Sánchez et al. [43] in cactus berry (*Myrtillocactus geometrizans*) fruits. However, the isobetanin bioaccessibility verified in the present study (46.1%) was higher than that obtained for cactus berry fruits [43].

In the same way as isobetanin, neobetanin showed 46% bioaccessibility at the end of gastrointestinal digestion. Overall, the bioaccessibility of betacyanins was 31.3%. The final total betacyanin content accessible for absorption by the human body was ≈ 9 mg/100 g fw of beetroot. According to previous reports, betalains are stable in a pH range of 3.5 to 7.0 (with greater stability at pH 5–6) [44], and lowering the pH to gastric conditions can led to decarboxylation of the compounds and cause their degradation [45]. A study carried out with betanin isolated from beetroot juice obtained a 35% decrease after gastric digestion due to impaired stability at an acidic pH of 2 [46]. However, in addition to the influence caused by the absence of a food matrix [42], the degradation mechanisms in gastric-like environments are still not entirely clear. On the other hand, the lower betanin degradation during IP may be due to the pH value [31]. Still, alkaline conditions induce hydrolysis of betacyanins by hydrolytic cleavage of the aldimine bond, leading to a decrease in color intensity due to the production of yellow and colorless degradation products [47]. Studies have also shown that the bioaccessibility of these natural pigments varies according to the food product and their concentration [42].

### 3.5. Antioxidant Activity

**Figure 5:**
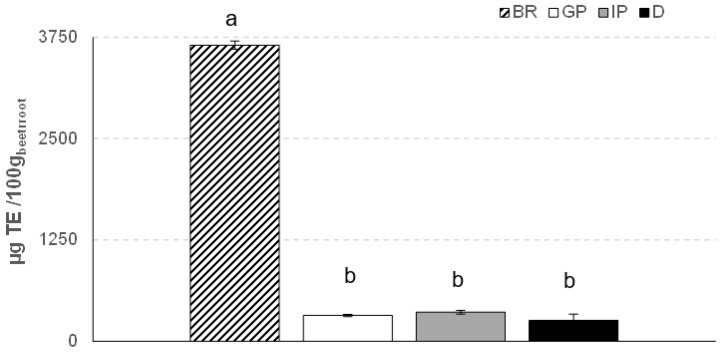
Antioxidant activity (µg TE/100 g fw of beetroot) of beetroot (BR), gastric phase (GP), intestinal phase (IP), and digested (D) sample. Different letters indicate significant differences (*p* < 0.05) between samples.

The antioxidant activity of undigested and digested beetroot powder samples was evaluated to elucidate its potential beneficial effects on human health. For this, an ex vivo bioassay was used to evaluate the ability of the samples to protect the RBC membrane from oxidative damage caused by the free radical generator added to the in vitro reaction system. Figure 5 shows the antioxidant activity results for the beetroot sample before and after each digestion phase. The significant (*p* < 0.05) decrease in antioxidant activity during in vitro gastrointestinal digestion was already remarkable in the gastric phase. These results suggest that the beetroot antioxidant compounds are unstable under gastrointestinal conditions. Significant losses in the antioxidant activity of fruit juices were also observed by other authors [48,49].

In this study, a statistical correlation analysis was performed to evaluate the contributions of the various identified compounds to the antioxidant activity of beetroot samples. Betacyanins played a major role in the antioxidant activity of the samples, showing the highest Pearson correlations coefficients, namely 0.9920 (*p* < 0.05), 0.9785 (*p* < 0.05), and 0.8472 (*p* < 0.05) for betanin, neobetanin, and isobetanin, respectively. The antioxidant action mechanisms of these biologically active compounds are known and can be found in the literature [50]. Strong relationships between the phenolic content and the antioxidant capacity of fruits and vegetables have also been shown in previous studies [51,52].

### 3.6. Contribution to Mineral Requirements

Beetroot is a nutrient-rich food that contributes to meeting the intake requirements for mineral elements and other micronutrients established by the current regulations [53]. The contribution to the dietary reference intake (DRI) of a given nutrient is defined based on its total content measured in the food product [53]. As shown in Table 2, the contribution of a 100 g serving of the studied spray-dried beetroot powder sample to the DRI of minerals (according to Regulation (EU) No 1169/2011) [53] is relatively low. The BR sample contributed 7.4% of the DRI of Fe (14 mg/day), 2.37% of the DRI of P (700 mg/day), and less than 1.5% of the DRI for the other elements, in the following decreasing order: K > Na > Mg > Ca.

This study showed that the amounts of minerals, organic acids, and betacyanins that are released from the food matrix and solubilized in the gastrointestinal tract for subsequent absorption by the intestinal epithelium vary during the in vitro simulated digestion. As shown in Figure 2, the bioaccessible fraction of mineral elements decreased during digestion. Therefore, the contribution of these micronutrients to the DRI decreased from 9–12% in the gastric phase, from 24–34% in the intestinal phase, and from 35–57% in the digested sample (Table 2). Thus, a 100 g portion of the digested sample still contributes about 3.4% of the DRI of Fe, a trace element that plays an important role in biologic processes involving oxygen transport and storage, as well as oxidative metabolism [55]. Despite this, the daily consumption of beetroot and derived food products, such as spray-dried beetroot powder and beetroot juice, could be an important strategy to provide a wide range of micronutrients and antioxidant compounds to the general population. Similar observations were made by other researchers when evaluating the bioaccessibility of mineral elements from tomato farmers’ varieties and their contribution to the DRI [56]. 

## 4. Conclusions

Although beetroot powder has a high content of bioactive compounds, such as betacyanins, the decrease in antioxidant activity during in vitro simulated gastrointestinal digestion stood out, suggesting that the antioxidant compounds of this root vegetable are unstable under such simulated physiological conditions. In the particular case of betanin (betanidin-5-*O*-glucoside), it was highly unstable during digestion, possibly due to its hydrophilic nature. The bioaccessibility of mineral elements after the entire process ranged from 43 to 65%, depending on the quantified mineral. Among these, the bioaccessibility of Mg was higher, while that of Ca and Fe was lower. A major release of organic acids during digestion was observed for quinic acid. Overall, these results contribute to understanding the potential fate of beetroot minerals, organic acids, and betacyanins in human nutrition, and will be useful for the development of improved formulations of beetroot-containing ingredients and food products. Although the contribution of a 100 g serving of the spray-dried beetroot powder to the DRI of mineral elements was relatively low, a daily consumption of beetroot and derived food products could be a strategy to provide a wide range of micronutrients (minerals and vitamins) and antioxidants (such as betacyanins) to the general population. In future studies, it will be important to evaluate the bioaccessibility of other beetroot constituents, including vitamins and sugars, as well as to identify the antinutritional factors that interfere with their bioaccessibility. Furthermore, the comparison of the results of this work with in vivo bioaccessibility data would be interesting.

## Figures and Tables

**Table 1:** Betacyanins identified in the beetroot powder sample. The retention time (Rt), wavelength of maximum absorption in the visible region (λ_max_), and mass spectral data are presented.

Peak	Rt (min)	λ_max_ (nm)	[M-H]^−^ (*m/z*)	MS^2^ (*m/z*)	Tentative Identification
1	17.4	533	551	389(100), 345(6), 150(8)	Betanidin-5-*O*-glucoside (betanin)
2	18.8	531	551	389(100), 345(7), 150(10)	Isobetanidin-5-*O*-glucoside (isobetanin)
3	22.8	462	549	389(100)	14,15-Dehydrobetanin (neobetanin)

**Table 2:** Contribution of the beetroot sample (BR) for the dietary reference intakes (DRI) of mineral elements, and also the contribution of the gastric phase (GP), intestinal phase (IP) and digested (D) samples (average per 100 g portion). For each element, the percentage decrease in contribution compared to the undigested sample (BR) is shown in parentheses.

Samples	P (% Contribution)	K (% Contribution)	Na (% Contribution)	Ca (% Contribution)	Mg (% Contribution)	Fe (% Contribution)
BR	2.37 ± 0.05	1.6 ± 0.1	1.46 ± 0.05	0.60 ± 0.05	1.39 ± 0.07	7.4 ± 0.4
GP	2.12 ± 0.03 (−10%)	1.45 ± 0.05 (−12%)	1.23 ± 0.01 (−16%)	0.51 ± 0.02 (−14%)	1.22 ± 0.04 (−12%)	6.7 ± 0.2 (−9%)
IP	1.79 ± 0.08 (−24%)	1.16 ± 0.09 (−29%)	1.02 ± 0.01 (−30%)	0.39 ± 0.02 (−34%)	1.06 ± 0.02 (−24%)	4.9 ± 0.4 (−34%)
D	1.24 ± 0.02 (−48%)	0.80 ± 0.07 (−51%)	0.74 ± 0.06 (−49%)	0.26 ± 0.02 (−57%)	0.90 ± 0.03 (−35%)	3.4 ± 0.2 (−54%)

RDI according to Regulation (EU) No 1169/2011: 700 mg/day for P; 2000 mg/day for K; 800 mg/day for Ca; 375 mg/day for Mg; and 14 mg/day for Fe [53]. For Na, 2000 mg/day is the level likely to allow most of the general population to maintain Na balance and for which there is sufficient confidence in a reduced risk of cardiovascular disease in the general adult population [54].

## Data Availability

Not applicable.

## Supplementary Materials

The following supporting information can be downloaded at: https://www.mdpi.com/article/10.3390/foods12020338/s1, Table S1: Phases of the method used for simulated gastrointestinal digestion of beetroot; Table S2: Method, equipment, and analytical conditions used in the analysis of minerals, organic acids and betacyanins; Figure S1: UFLC profile of commercial organic acid standards recorded at 215 nm. Peak identification: 1-oxalic acid; 2-quinic acid; 3-malic acid; 4-shikimic acid; 5-citric acid; 6-succinic acid; and 7-fumaric acid; Figure S2: Maximum UV-vis absorption spectrum (recorded at 215 nm) of oxalic acid (A), quinic acid (B), and malic acid (D). Figure S3: UFLC organic acid profile of beetroot (A), gastric phase (B), intestinal phase (C), and digested (D) sample, recorded at 215 nm. Peak identification: 1- oxalic acid; 2-quinic acid; and 3-malic acid; Figure S4: HPLC chromatographic profile of betacyanins in beetroot (A), gastric phase (B), intestinal phase (C), and digested (D) sample recorded at 535 nm. Peak identification: 1-betanidin-5-*O*-glucoside (betanin); 2-isobetanidin-5-*O*-glucoside (isobetanin); and 3-14,15-dehydrobetanin (neobetanin); Figure S5: Maximum absorption spectrum (recorded at 535 nm) and mass spectrum (full MS and MS^2^), obtained by HPLC-DAD-ESI/MS*^n^*, of the three betacyanins identified in the beetroot samples. Peak identification: 1-betanidin-5-*O*-glucoside (betanin); 2-isobetanidin-5-*O*-glucoside (isobetanin); and 3-14,15-dehydrobetanin (neobetanin).
