# Supplementary material for: The In Vitro Simulated Gastrointestinal Digestion Affects the Bioaccessibility and Bioactivity of Beta vulgaris Constituents
Source: Foods. 2023 Jan 11;12(2):338. doi: 10.3390/foods12020338 (PMC9857886; doi:10.3390/foods12020338)
Supplement: Supplementary file 1 [file foods-12-00338-s001.zip › foods-2085071-supplementary.pdf]

## Supplementary Material

# The *In Vitro* Simulated Gastrointestinal Digestion Affects the Bioaccessibility and Bioactivity of *Beta vulgaris* Constituents

Marta Igual, Ângela Fernandes, Maria Inês Dias, José Pinela, Purificación García-Segovia, Javier Martínez-Monzó, Lillian Barros

**Table S1.** Phases of the method used for simulated gastrointestinal digestion of beetroot.

| Phases | Conditions |
| --- | --- |
| Oral phase (GP) | The sample was mixed with simulated salivary fluid (1:1) and amylase (75 U/mL) at pH 7 for 2 min. |
| Gastric phase (GP) | The oral bolus was mixed with simulated gastric fluid (1:1), pepsin (2,000 U/mL) and gastric lipase (60 U/mL) at pH 3 for 2 h. |
| Intestinal phase (IP) | The gastric chime was mixed with simulated intestinal fluid (1:1) and pancreatin (Trypsin activity 100 U/mL) at pH 7 for 2 h. |
| Digested (D) sample | The mixture was centrifugated at 4500 rpm for 30 min and then filtered through a 1 μm glass-fiber membrane. |

**Table S2.** Method, equipment and analytical conditions used in the analysis of minerals, organic acids and betacyanins.

| | **Minerals** | **Organic acids** | **Betacyanins** |
|---|---|---|---|
| **Method** | Inductively coupled plasma optical emission spectroscopy (ICP-OES). | Ultra-fast liquid chromatography with photodiode array detection (UFLC-PDA). | High-performance liquid chromatograph with diode array detection/electrospray ionization mass spectrometry (HPLC-DAD-ESI/MS). |
| **Equipment & Analytical Conditions** | An inductively coupled plasma optical emission spectrometer (700 Series ICP-OES; Agilent Technologies, Santa Clara, United States) equipped with an axial viewing and a charge-coupled device detector with a radiofrequency generator of 40 MHz, plasma gas flow rate of 15 L/min, auxiliary gas flow rate of 1.5 L/min, power of 1 kW and nebulizer gas (One Neb 2) pressure of 200 kPa. | An UFLC system (Shimadzu Corporation, Kyoto, Japan) with photodiode array detection (PDA) equipped with a SphereClone reverse-phase C18 column (5 µm particle size, 250 × 4.6 mm) (Phenomenex, Torrance, CA) thermostated at 35 °C was used for compounds separation. The mobile phase consisted of a sulfuric acid solution (3.6 mM) at a flow rate of 0.8 mL/min. | An HPLC (Dionex Ultimate 3000 HPLC, Thermo Scientific, San Jose, CA, USA) system and a Linear Ion Trap LTQ XL MS (Thermo Finnigan, San Jose, CA, USA) MS equipped with an ESI source. Separation was performed on a Waters Spherisorb S3 ODS-2 C18 column (3 µm, 4.6 mm × 150 mm, Waters, Milford, MA, USA) thermostatted at 35 °C. The solvents ((A) 0.1% trifluoroacetic acid (TFA) in water, (B) acetonitrile) were used as folows: 10% B for 3 min, from 10 to 15% B for 12 min, 15% B for 5 min, from 15 to 18% B for 5 min, from 18 to 30% B for 20 min, from 30 to 35% B for 5 min, and from 35 to 10% B for 10 min. |

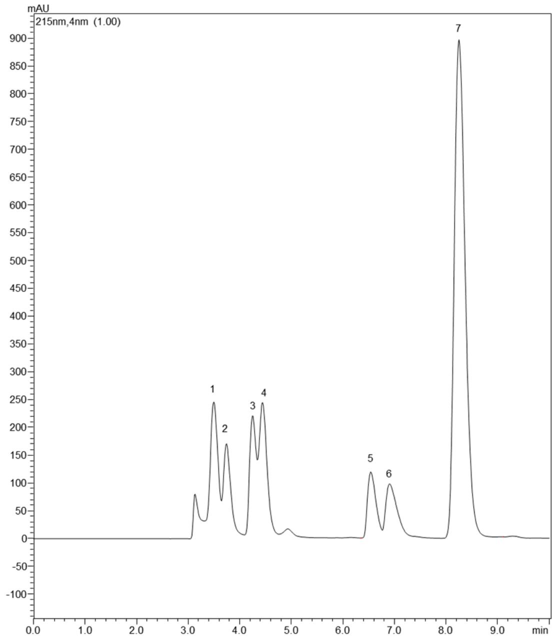


**Figure S1**. UFLC profile of commercial organic acid standards recorded at 215 nm. Peak identification: 1- oxalic acid; 2- quinic acid; 3- malic acid; 4- shikimic acid; 5- citric acid; 6- succinic acid; and 7- fumaric acid.

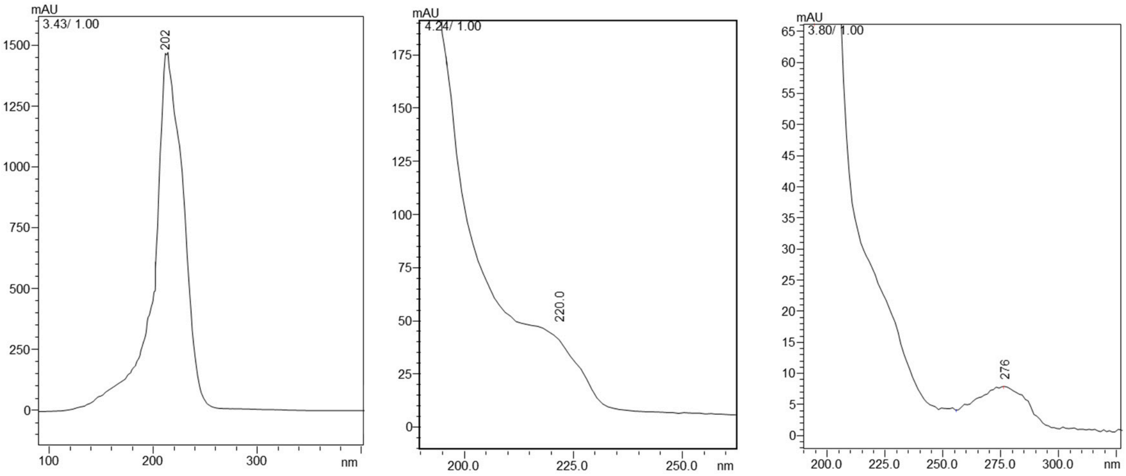


A B C

**Figure S2**. Maximum UV-Vis absorption spectrum (recorded at 215 nm) of oxalic acid (A), quinic acid (B), and malic acid (D).

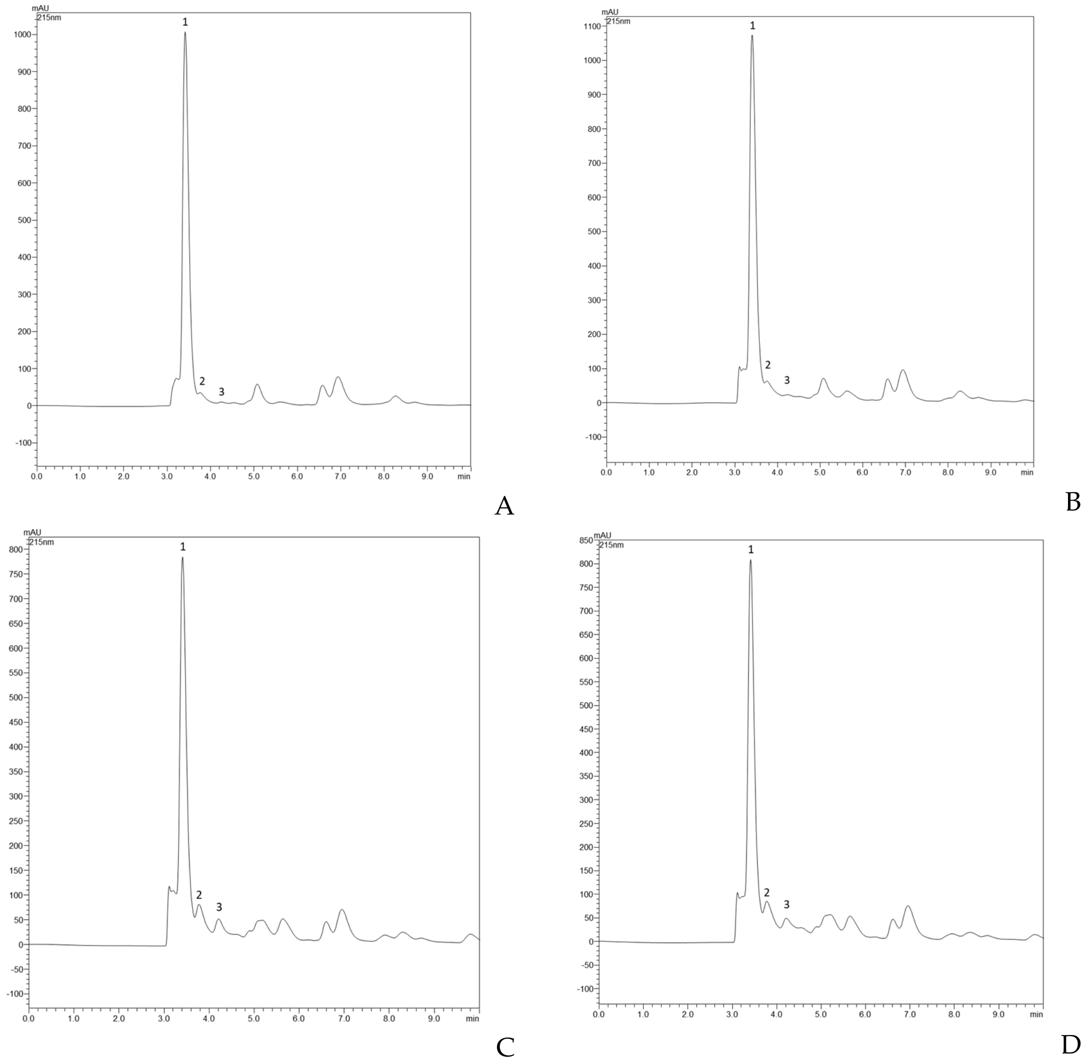


**Figure S3**. UFLC organic acid profile of beetroot (A), gastric phase (B), intestinal phase (C), and digested (D) sample recorded at 215 nm. Peak identification: 1- oxalic acid; 2- quinic acid; and 3- malic acid.

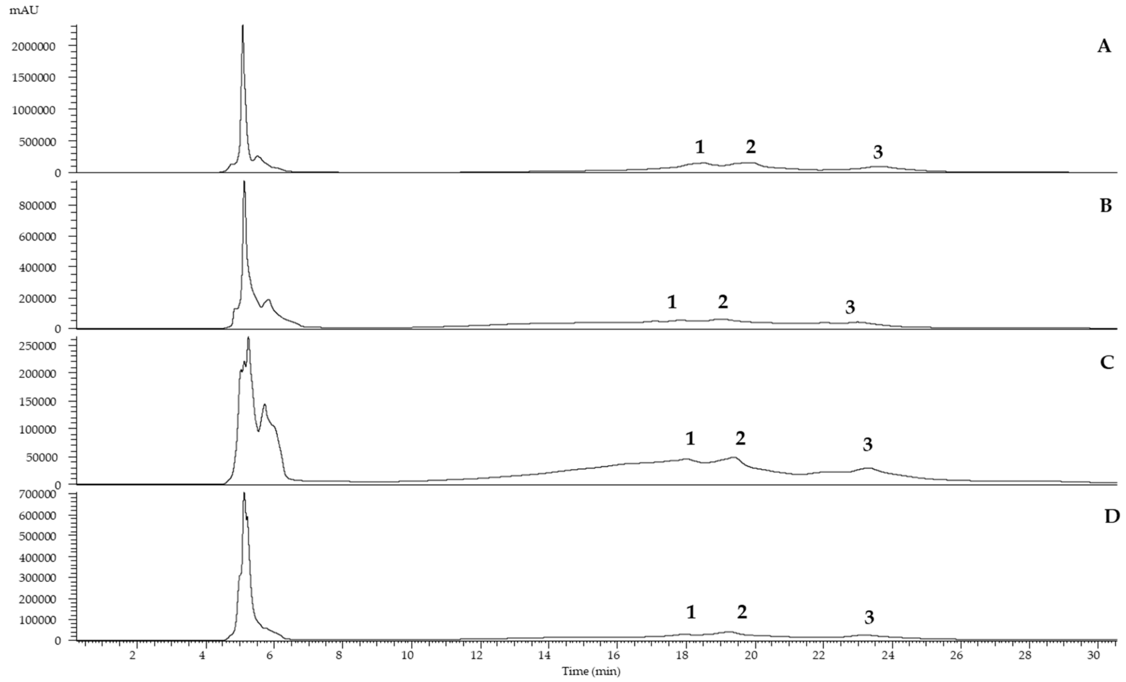


**Figure S4**. HPLC chromatographic profile of betacyanins in beetroot (A), gastric phase (B), intestinal phase (C), and digested (D) sample recorded at 535 nm. Peak identification: 1- betanidin-5-*O*-glucoside (betanin); 2- isobetanidin-5-*O*-glucoside (isobetanin); and 3- 14,15-dehydrobetanin (neobetanin).

| Peak | UV-Vis Spectra | Mass Spectra: Full MS | Mass Spectra: MS² |
|---|---|---|---|
| 1 | 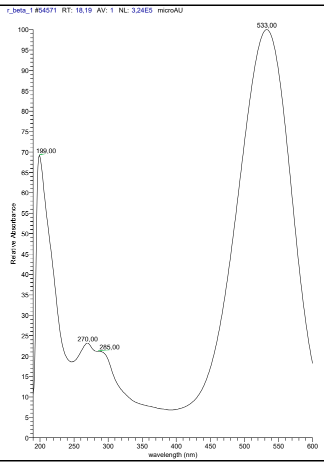 | 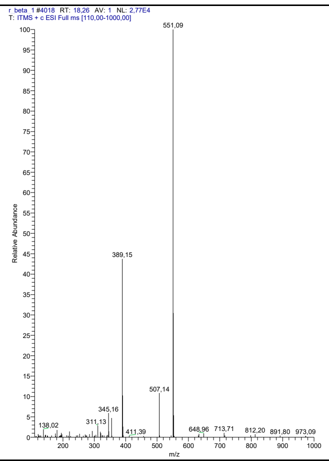 | 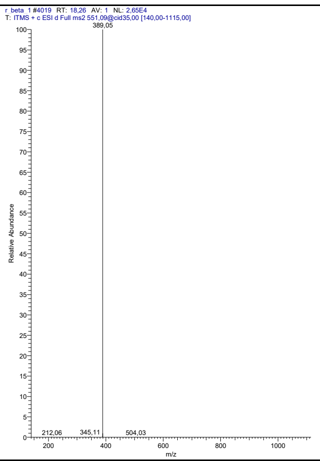 |
| 2 | 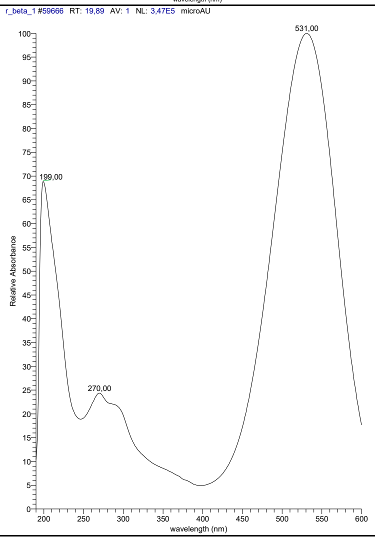 | 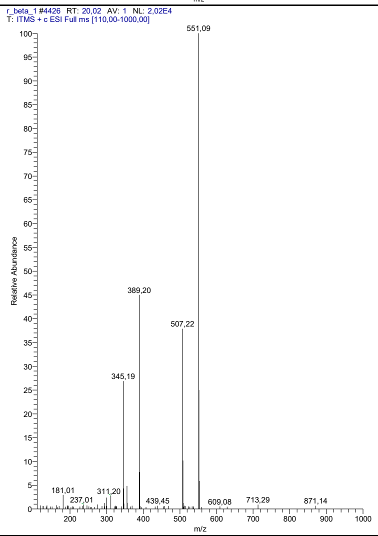 | 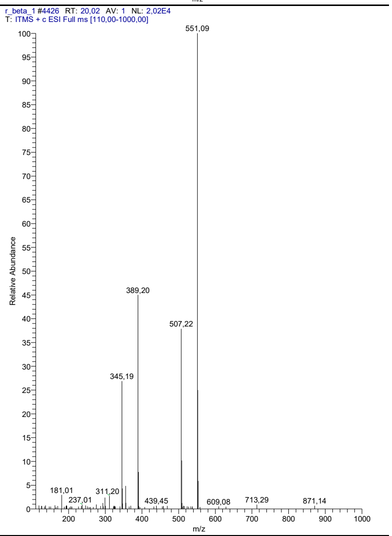 |
| 3 | 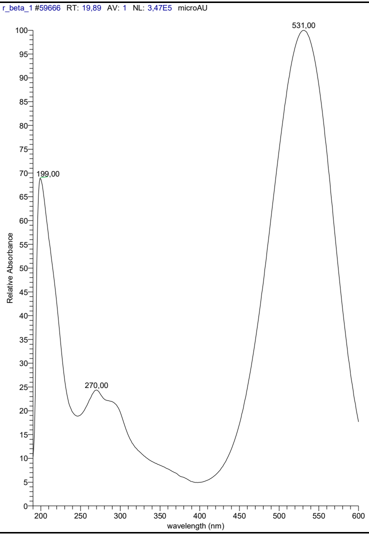 | 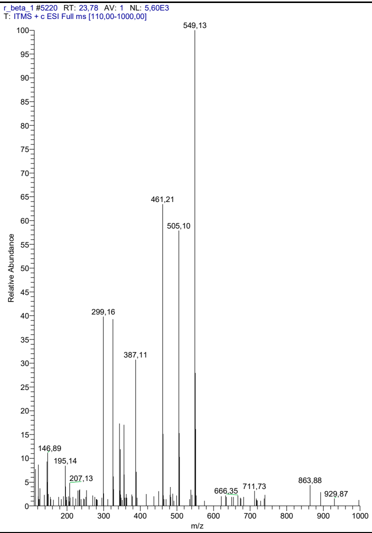 | 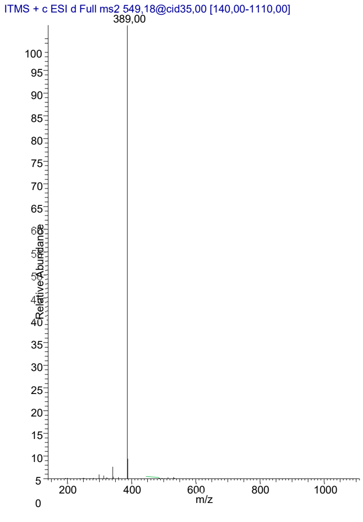 |

**Figure S5**. Maximum absorption spectrum (recorded at 535 nm) and mass spectrum (full MS and MS²), obtained by HPLC-DAD-ESI/MS$^n$, of the three betacyanins identified in the beetroot samples. Peak identification: 1- betanidin-5-*O*-glucoside (betanin); 2-isobetanidin-5-*O*-glucoside (isobetanin); and 3- 14,15-dehydrobetanin (neobetanin).
